# Predictors of Long-Term Outcome in STEMI and NSTEMI—Insights from J-MINUET

**DOI:** 10.3390/jcm9103166

**Published:** 2020-09-30

**Authors:** Ivan Lechner, Martin Reindl, Bernhard Metzler, Sebastian J. Reinstadler

**Affiliations:** University Clinic of Internal Medicine III, Cardiology and Angiology, Medical University of Innsbruck, Anichstrasse 35, A-6020 Innsbruck, Austria; Ivan.Lechner@tirol-kliniken.at (I.L.); Martin.Reindl@tirol-kliniken.at (M.R.); Bernhard.Metzler@tirol-kliniken.at (B.M.)

Although patients with ST-segment elevation myocardial infarction (STEMI) and non-ST- segment elevation myocardial infarction (NSTEMI) share similar risk factors and comparable pathophysiology [1,2], short and long-term outcomes differ considerably. While patients after STEMI have a higher in-hospital mortality rate and worse short-term outcome, NSTEMI patients have poorer long-term prognosis [3,4,5,6]. As yet, the underlying causes that explain this phenomenon are not fully understood, but various pathomechanisms have been proposed. Patients presenting with NSTEMI have a worse clinical risk profile (i.e., are significantly older, have a higher burden of comorbidities and a more frequently history of coronary artery disease) [3,5], higher rates of recurrent ischemia [3], and are less likely to receive guideline-recommended treatment strategies at discharge [5,6,7].

In this issue of the *Journal of Clinical Medicine*, Toyoda et al. [8] provide some new insights into the prognostic stratification and long-term outcomes of patients with NSTEMI and STEMI, by performing a post hoc analysis of the J-MINUET (Japanese Registry of Acute Myocardial Infarction Diagnosed by Universal Definition)-Study. This large Japanese-wide multicenter registry enrolled 3283 consecutive patients with acute myocardial infarction (AMI), from 28 medical institutions between July 2012 and March 2014 [4,9]. In the present study, the authors evaluated independent predictors of long-term outcome in patients after AMI, by using Cox proportional hazard models and by proposing specific risk score models. For this purpose, the authors evaluated a total of 111 baseline demographics and clinical characteristics [9]. According to the authors, in Japan, creatine kinase (CK)-based criteria are still widely used in the current clinical setting. Therefore, they focused specifically on the following three groups of patients: STEMI (68.9%), NSTEMI with creatine kinase elevation (NSTEMI+CK (17.1%)) and NSTEMI without creatine kinase elevation (NSTEMI-CK (14.0%)). Prognostic factors varied widely among all three subgroups, and long-term outcomes were not only worse in NSTEMI+CK, but also in NSTEMI-CK, when compared to STEMI patients. One might therefore speculate on whether it would be useful to differentiate between AMI diagnosed by CK-based definitions and AMI diagnosed by troponin-based definitions.

The authors were able to demonstrate this in this well-conducted post hoc analysis, with the following strengths. This includes data availability from a prospective multicentric observational study with a large sample size (*n* = 3283), observation period of 3 years, and relevant clinical endpoints [4,9]. These findings provide novel insights in AMI pathophysiology by adding information, such as the different prognostic factors, not only between STEMI and NSTEMI, but also in NSTEMI+CK and NSTEMI-CK elevation. In addition, the investigators propose risk-scores to predict long-term prognosis in patients after AMI, which consequently could be a step towards personalized risk-calculation, especially in patients at an increased risk. This is also of interest, as several studies have shown that NSTEMI patients are less likely to receive guideline-recommended treatment strategies and less frequently participate in cardiac rehabilitation programs [5,6,7].

However, in the current paper by Toyoda et al. [8], there are several points that require consideration. First of all, this represents a post hoc analysis with all inherent limitations of such a study design. Another relevant drawback is the lack of any internal or external validation. The findings have therefore to be considered as hypothesis-generating. In particular, the conclusions for patients with NSTEMI should be considered with caution, as they were divided into two subgroups and made up only a minority of the study population. Another important issue is the principal question of the appropriateness of stratification of NSTEMI patients by using CK [10]. The present study was conducted at a time at which CK (or more precisely creatine kinase-myocardial band) measurements was a possible alternative to troponin measurements [11]. However, there is now a general consensus that CK determination has no additional benefit for diagnosing an acute myocardial infarction [10]. The differences in outcome between CK+ and CK- NSTEMIs are not unexpected, as CK correlates with infarct size with a similar correlation coefficient as troponin [12], and therefore differences in myocardial injury likely contribute to these differences. Another notable limitation of this post hoc analysis is the fact that no predefined variable selection was conducted. The authors included all clinical available parameters in risk-score-calculation, hence, resulted in some implausible mechanisms, which was also discussed by the authors [8]. Therefore, these results have to be interpreted with caution.

Lastly, it has to be considered that the study cohort was relatively inconsistent with both type 1 and type 2 infarction included. NSTEMI patients as well as type 2 infarction is more commonly found in the elderly population and in patients who have a higher burden of comorbidities, resulting in poorer prognosis [3,5,13]. This could be a possible explanation for adverse outcome in NSTEMI-CK patients, and could be an important bias of outcome prediction.

To summarize, the authors were able to provide novel insights into AMI prognostication, and could corroborate that the prognostic factors differ considerably among patients with STEMI and NSTEMI, but also in NSTEMI+CK and NSTEMI-CK. The optimal, biomarker-based, the stratification of patients after AMI needs further validation and research.
